# Uric Acid as a Factor of Disease

**Published:** 1895-11

**Authors:** E. Van Goidtsnoven

**Affiliations:** Atlanta, Ga.


### URIC ACID AS A FACTOR OF DISEASE.

BY E. VAN GOIDTSNOVEN, A.M., M.D.,

ATLANTA, GA.

I doubt whether in the catalogue of etiological factors, one can be found more common, more insidious in its evil-doing, more metastatic in its manifestations, more hydra-headed in its stubborn resistance to treatment, more lethal in its results, and yet more ignored by the medical profession, than uric acid.

Every stage of life, every condition of existence, is susceptible of lithemia. We can scarcely conceive of a disorder which, having baffled all attempts at research and treatment, is not suggestive or evocative of this pathological causation.

The infant, the adolescent, the adult, the senile, the rich, the poor, the landlord, the peasant, the banker, the mechanic, the profession, the laity, all are subject to imperfect elimination, deficient excretion and consequent diathetic or cachectic lithemia.

Urea is, so to speak, the last hystolitic product, the *ultima thule* of tissue decay which, incapable of being more highly organized, can no longer serve the purposes of life, and is therefore secreted by the urine.

Uric acid has many congeneric associates, such as xanthine, hypoxanthine, creatine, creatinine, tyrosine. They present many points of resemblance. Their chemical formula being almost identical, their relationship is closer than that of an isomeric nature. All are early excreta of tissue decay, constitute an array of powerful neurotic poisons, and when not removed from the organism through the instrumentality of katabolic agencies, and the media of excretory organs, produce serious disorders, often ending in coma, convulsions and death.

It is now a well known fact that the formation of uric acid is antecedent to that of urea, that it occupies an intermediate position between the proteid compounds as they enter the system as nutrients and their final elimination into urea.

An excess of uric acid produces toxic phenomena, perversion of function and organic disorders throughout the whole economy.

To those who recognize a clinical and pathological relation between lithemia and rheumatism, gout, eczema, asthma, dyspepsia, diabetes, Bright's disease, etc., etc., it is somewhat amazing that the majority of the medical profession, and especially the insurance examiners, should restrict the importance of urine analysis to diabetic and albuminous excreta.

We all admit that nitrogenous waste creates a condition in the organism incompatible with health, and that blood loaded therewith becomes the seat of chemical changes which materially differ from those ordinarily taking place in its normal constituents. The particular chemical compounds formed under these circumstances are not readily excreted. Remaining in the blood, they accumulate, and minute bioplasts or leucocytes grow and multiply. At length an influence upon the nerves is exerted, and there ensue toxic phenomena, toxic manifestations of diseases which are auto-genetic, auto-infectious, and frequently septic. Impaired vision, neuralgia, hypochondriasm, gout, rheumatism, eczema, dyspepsia, bronchorrhea, bronchitis, colds, catarrhs of mucous membrane, diarrhea, diabetes mellitus and insipidus, many forms of supposed gout, such as valvulitis, prostatitis, and even testitis. All these disorders can, in many instances, be traced to the presence of toxic by-products in the organism, and especially of uric acid.

It is generally believed that nitrogenized waste passes off by the kidneys only. We should remember, however, that there is such a thing as metastasis of excretion; that the skin, the lungs, the bowels are emunctories of no less importance than the renal organs; that there exists between these excreting organs a complementary relation, and that if the function of one be interfered with, another performs it, to a certain extent. These are not diseases *per se*. They simply indicate that one organ is called upon to perform the duties of another organ temporarily affected with inadequacy or insufficiency. But in truly pathological conditions these compensatory actions and vicarious functions are increased to a burthensome degree and lead to gross disorders. Hence, we readily understand such outcome as above enumerated when uric acid, which is insoluble in the fluids of the body, is allowed to cumulate and remain within the economy, especially if there be hepatic or renal inadequacy.

I now come to a question which I consider of practical interest. How are we to detect the presence of uric acid and its toxic associates in the animal tissues?

Whenever I am called to treat a patient complaining of any of the disorders above mentioned, if previously intractable, I prescribe terebene (McK. & R.) in fifteen-drop doses, to be triturated in sugar and administered every four hours. When the patient has taken from one to two drachms of terebene, I examine the urine, and the murexide test almost invariably *tells the tale.*

According to Dujardin-Beaumetz, terebinthinates introduced into the system, whether by the skin, lungs, or stomach, enter the general circulation. They then decompose, and while the volatile substances are eliminated by the lungs, the fixed parts are excreted by the urine and exercise their individual properties upon the urinary passages, *provided the latter be in a permeable state.*

In health, and even in many a morbid condition, terebinthinates impart a characteristic odor to the urine—that of violets. In nephritic patients, however, these substances, when administered, gradually fail, with the progress of disease, to give rise to this odor. De Beauvais calls attention to the want of elimination of this odor in the last stage of Bright's disease. Terebene is the terebinthinate *par excellence.* I use it:

First, as the best uric eliminator at my command, and therefore I value it as a therapeutic agent of the highest order.

Secondly, as an infallible indicator and enunciator of lithemic condition.

Thirdly, as a guide whose assistance is of considerable value in studying, ascertaining, and appreciating the successive stages of Bright's disease.

For more than five years I have given this subject considerable thought, study, and practical application. I now submit these views and suggestion to the profession in the hope that they will elicit consideration and no little criticism. If there be chaff therein, let it be blown off. I shall be but too happy if only a few grains remain in the winnowing van *pro bono publico.*

I append the report of a few cases. I will begin with my own case.

Case I. I am a sufferer of "hay fever"; have been so for many years, and am satisfied it is a disease of microbic origin, and that there is no cure for it unless it be through immunition, which, thus far, and for obvious reasons, I have not as yet been subjected to. One of the characteristics of this disorder, attending especially its last stage, is dyspnea. I frequently apply to my friends, Drs. Brown & Campbell, of this city, for relief, and never fail to obtain it. One of their forms of treatment is inhalation of terebene. At the very incipiency of this treatment, I felt that the inhaling process was immediately followed by relief. Normal respiration was restored for a considerable length of time. Diaphoresis simultaneously ensued, and the next micturition revealed a free deposit of "brick dust" sediment, not unlike the fever excreta of physicians, together with the strong odor of violet. Examination of the urine with the murexide test, elicited the unmistakable presence of uric acid. Each successive treatment was followed by the same temporary relief, the same condition and characteristics of urine; and I became convinced that dyspnea, as a secondary lesion, in my case, was due to the presence of uric acid, and that terebene promotes the elimination of the same through the dialyzing membrane of Bowman's capsule.

Case II. J. C. T., infant at the breast; age three months; of German parents, both *heavy* beer-drinkers. This child passed but a small quantity of urine, strongly ammoniacal, at long intervals, each micturition accompanied by violent pain along the urinary tract. This case had resisted all forms of treatment previous to my being called. Analysis detected uric acid in abundance. Diagnosis: Lithemia from hereditary transmission.

*Treatment.*—Emulsion of terebene. Relief very prompt.

CASE III. H. F. M., German; age 73; an old Confederate soldier. Drinks whisky rather freely. Arthritic cachexia from exposure to cold, fatigue and privations during the late unpleasantness. Amaurosis, cardiac hypertrophy. At first, no puffing of lower eyelids, no dropsy of lower extremities. Intense burning sensation around the thighs and in the bowels; liver cirrhotic; dyspnea; urine copious, pale; albumen trifling; "brick dust" deposit small, of fine, granular casts; specific gravity 1005. Analysis shows uric acid.

*Treatment.*—Terebene in twenty-drop doses, four times a day. Result: At first, disappearance of dyspnea and stinging sensation. Odor of violet well pronounced. Increase of uric deposit. Subsequently gradual diminishing of sediment and of characteristic odor; also of micturition. Wasting rapid. Meanwhile, puffing of lower eyelids, and swelling of lower limbs intense. Patient dies, evidently, of that form of Bright's disease designated as chronic interstitial nephritis.

CASE IV. Reverend C., a prominent clergyman of this State. Found in a state of insensibility on the doorsteps of a church; brought to a friend's residence. When first seen by me, he had somewhat recovered his senses. Hebetude, however, was well marked, pupils dilated, pallor, ammoniacal breath and perspiration, pulse imperceptible, great dyspnea, nervous twitchings, leading me to diagnose uremia. Had yellow fever and dengue years ago. Cirrhosis of the liver as a result. Urine scanty and loaded with uric acid. Blood evidently poisoned by urine constituents.

*Treatment.*—First administered a brisk cathartic. Then prescribed terebene in twenty-drop doses every three hours. Urine heavily loaded with "brick dust" deposits for the following ten days. A marked improvement then gradually took place. The dyspnea and hebetude finally disappeared, together with the excreta.

CASE V. Paul G., age 23; acute articular rheumatism, involving every joint. Excruciating pain and tenderness. Serous effusion, shifting from one articulation to the other. Temperature 105. Pulse 120.

Examination of urine at first revealed no uric acid.

*Treatment.*—Free catharsis and terebene. This obtained a prompt relief. The uric deposits were very free for several days, and finally disappeared, together with the disorder.

CASE VI. K. C., age 64. Inflammation of the prostate gland. Attacks periodic and lasting about a week. Had a severe attack of gout at the age of 45. Chalk stone deposits around the big toes and in the left ear. We all know that uric acid is always present and in large excess in the blood of gouty sufferers.

*Treatment.*—Terebene in doses as above stated. Relief very prompt. At each recurrence of the trouble the patient resumes the same treatment, unaided by me, with the same happy effect.

CASE VII. Miss U. V., age 35. A stout, tall, portly looking lady, florid complexion. Had malarial fever and pneumonia about ten years ago. Has been afflicted with dyspnea ever since that time. About five weeks ago had a severe attack of grippe, for which I prescribed the following:

| | | |
|---|---|---|
| ℞ | Codeinæ sulphatis | gr. i. |
| | Ammonolis | ʒ i. |
| | Quinæ sulphatis | ʒ ss. |
| | Caffeinæ citratis | gr. xii. |

M. Div. in caps. xii.

S. One (1) capsule every four (4) hours.

Also:

| | | |
|---|---|---|
| ℞ | Kalii iodidi | gr. 80 |
| | Ammonii chloridi | gr. 160 |
| | Misturæ liquirrhizzi comp | f ℥ viii. |

M. S. One (1) tablespoonful every four (4) hours.

Alternating in such a way that medicine be administered every two (2) hours.

This treatment proved very satisfactory. All the leading symptoms of grippe disappeared in less than a week. I dismissed the patient on the tenth day. I was sent for again on the following day, when I found my patient suffering with intense "backache." Urine was scanty and heavily loaded with "brick dust" deposit. Analysis revealed uric acid. Dyspnea had greatly increased—in fact, alarmingly so. I prescribed terebene in twenty-drop doses every three hours. This was followed by complete relief in thirty-six hours.

